# The Road Not Taken: Neural Correlates of Decision Making in Orbitofrontal Cortex

**DOI:** 10.3389/fnins.2012.00131

**Published:** 2012-09-11

**Authors:** Adam P. Steiner, A. David Redish

**Affiliations:** ^1^Graduate Program in Neuroscience, University of MinnesotaMinneapolis, MN, USA; ^2^Department of Neuroscience, University of MinnesotaMinneapolis, MN, USA

**Keywords:** orbitofrontal cortex, covert representation of reward, regret, counterfactual, vicarious trial and error, multiple T

## Abstract

Empirical research links human orbitofrontal cortex (OFC) to the evaluation of outcomes during decision making and the representation of alternative (better) outcomes after failures. When faced with a difficult decision, rats sometimes pause and turn back-and-forth toward goals, until finally orienting toward the chosen direction. Neural representations of reward in rodent OFC increased immediately following each reorientation, implying a transient representation of the expected outcome following self-initiated decisions. Upon reaching reward locations and finding no reward (having made an error), OFC representations of reward decreased locally indicating a disappointment signal that then switched to represent the unrewarded, non-local, would-have-been rewarded site. These results illustrate that following a decision to act, neural ensembles in OFC represent reward, and upon the realization of an error, represent the reward that could have been.

## Introduction

Several studies have postulated that the orbitofrontal cortex (OFC) generates predictions about rewards and facilitates re-evaluation when prior cues lead to a new outcome (Schoenbaum et al., 1998, 2003; Gottfried et al., 2003; Wallis and Miller, 2003; Padoa-Schioppa and Assad, 2006, 2008; McDannald et al., 2011; Lucantonio et al., 2012); however, most research in OFC has focused on decision making following overt, instructive cues indicative of reward (Schoenbaum and Eichenbaum, 1995a; Schultz et al., 1997; Gallagher et al., 1999; Tremblay and Schultz, 1999; Padoa-Schioppa and Assad, 2006; Hare et al., 2009; McDannald et al., 2011). In cued-response tasks, orbitofrontal neurons show increased firing to cues that have come to predict rewards (Schoenbaum and Eichenbaum, 1995a; Tremblay and Schultz, 1999; van Duuren et al., 2009). A very few studies have identified a role for OFC in decision making in the absence of explicit cues (Young and Shapiro, 2011).

When faced with a difficult decision, rats and humans sometimes pause and orient back-and-forth toward options or paths, a behavior termed vicarious trial and error (VTE; Muenzinger, 1938; Tolman, 1939; Johnson and Redish, 2007; Krajbich et al., 2010; Papale et al., 2012). During VTE and similar behaviors, neural signals in hippocampus and ventral striatum show evidence of covert decision-making processes (Johnson and Redish, 2007; van der Meer and Redish, 2009; van der Meer et al., 2010). Here we report that neural representations of reward in the OFC of behaving rats increased following VTE events at a decision-point, implying representation of the expected rewards during an internal, self-initiated decision.

When faced with outcomes that do not match expectations, human subjects report feeling disappointment (Camille et al., 2004; Chua et al., 2009). Economically, disappointment is defined as receiving less value than expected (Bell, 1985; Loomes and Sugden, 1986; Redish et al., 2007). When human subjects reported feeling disappointment, activity in OFC increased (Chua et al., 2009). Here, we report that, at reward locations on error trials, when no reward was present, neural representations of reward in OFC of behaving rats decreased, indicating a neural correlate of disappointment.

However, when better alternatives were known to be available, human subjects reported feeling regret (Camille et al., 2004). When human subjects reported feeling regret, neural activity increased in OFC (Coricelli et al., 2005). This realization that reward would have been received had an alternative action been taken can be defined as the *counterfactual* (Camille et al., 2004; Coricelli et al., 2005). In primates, OFC neurons have been shown to represent hypothetical alternative outcomes (Abe and Lee, 2011). Here, we report that, when faced with a lack of delivered reward (disappointment) after making a decision (implying the potential for regret), neural representations in rat OFC switched from encoding the local, unrewarded site, to encoding the non-local would-have-been rewarded site, representing a neural signal of the counterfactual necessary for regret.

In summary, following a decision to act, neural ensembles in OFC represent the expectation of reward, potentially guiding future evaluative processes, and upon the realization of an error, represent the reward that could have been.

## Materials and Methods

### Animals

Four Fisher Brown Norway rats aged 10–12 months at the start of behavior were used in this experiment. Prior to training, rats were handled for 2 weeks. On the last 6 days of the 2-week period, normal Teklad pellets were replaced with flavored pellets within the rats’ home cage. Rats had access to 15 g of white (unflavored), fruit-flavored, or banana-flavored food pellets, presented in random order during handling. Each flavor was presented once per day no more than twice during the 6-day sequence. Rats were housed on a 12-h light/dark cycle and training/probe trials occurred during the same time each day. During testing, rats were maintained at roughly 85% of their free feed weight. Rats had access to water at all times. All training procedures were in accordance with the National Institutes of Health guidelines and approved by the Institutional Animal Care and Use Committee at the University of Minnesota.

### Behavior: The multiple-T-LRA task

**Figure 1:**
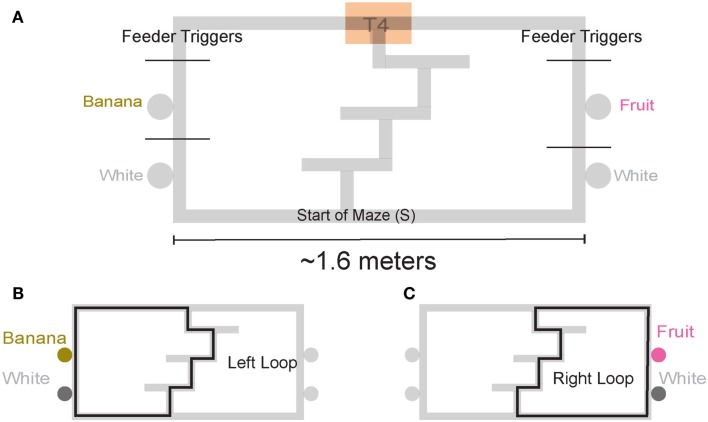
**Multiple T-LRA task behavior. (A)** Rats were placed at the start of the maze (S). Reward was delivered as animals crossed the reward-trigger lines. Reward trigger zones were spatially defined and did not vary from day to day. On any given the day, if the rat chose correctly, once he passed into the zone, pellets were delivered. Each side had two feeders, one flavored, one unflavored. Flavors at reward sites remained constant across all sessions. **(B)** Leftward maze loop. **(C)** Rightward maze loop.

We trained four rats on a continuous loop, multiple choice, maze task (Figure 1A). Reward was delivered under Left (L), Right (R), or Alternation (A) schedules (MT-LRA; Gupta et al., 2010; Blumenthal et al., 2011). The Multiple-T maze consisted of a figure-8 topology, with a central *navigation sequence* leading to a high-cost *choice point*. The choice point led to two, different *return rails*. Each had two feeders (Med-Associates, St. Albans VT, USA) and potentially provided 2 mg × 45 mg food pellets (Research Diets, New Brunswick, NJ, USA) each. The navigation sequence consisted of three low-cost choice points, at which the rat could turn around if he made a wrong choice. After a choice at the high-cost choice point at the end of the navigation sequence, the rat had to continue down the return rails before coming around for another lap. The left return rail provided banana-flavored pellets at the first feeder site, and unflavored (white) pellets at the second feeder site (Figure 1B) the right return rail provided fruit-flavored pellets at the first feeder and unflavored pellets at the second feeder site (Figure 1C). During training, if a rat tried to run backward on the navigation sequence or backward from the second feeder to the first feeder on one of the return rails or from one feeder side to the other across the top rail, the rat’s path was blocked by the experimenter with a PVC pipe. However, by the recording sessions, rats never turned around and did not need to be blocked.

The navigation sequence remained constant within a day, but changed from day to day. Whether reward was provided on a return rail or not depended on the choices made by the rat. Three reward contingencies were used: (L) turn left for reward, in which the left return rail always provided reward and the right did not, (R) turn right for reward, in which the right return rail always provided reward and the left did not, and (A) alternate for reward, in which the return rail not previously visited was rewarded. In the alternation (A) contingency, the first return rail visited was always rewarded on a given day. All reward site locations and flavors at each reward site were constant across all sessions. On a correct lap, reward was always presented. On error laps, reward was never presented.

Rats ran one 40 min session per day. Contingencies were presented in a pseudorandom order across days. The rat did not receive any cues informing it of the rewarded contingency. On each day, the rat was placed at the start of the maze and allowed to run through the navigation sequence and turn left or right at the final choice point for reward, but it did not know which contingency it faced. Rats were trained for an average 24 days on this task before surgery, until they were performing all three contingencies (L, R, and A) reliably.

Following surgery, rats were allowed to recover for 2–4 days, during which they had free access to food and water. After 2–4 days, rats were returned to the Multiple-T-LRA task. Recordings commenced when the rats returned to running a number of laps comparable to pre-surgery. To acclimate to the additional weight of the tether and hyperdrive implant before the probe sequence began, the rats were trained for several more days while tetrodes were advanced to target sites.

Following adaption to the increased running weight and achievement of large ensemble sizes, rats began the 6-day probe sequence. A probe day entailed a change in contingency after 18–22 min. Thus, the rat faced one of the three contingencies (left, right, or alternation) for ∼20 min, and then faced a new contingency for the second 20 min. Rats were not removed from the maze at the switch, nor were they signaled as to the switch. During probe sessions, the fourth T was always aligned to the middle of the top rail. This ensured that the path length from the high-cost choice point did not change to either the left return rail or the right return rail. We ran six probe days: *left/right*, *right/left*, *left/alternation*, *right/alternation*, *alternation/left*, and *alternation/right*. Each rat saw all six probe days, but the order of the six probe days was randomized between rats.

### Surgery

**Figure 2:**
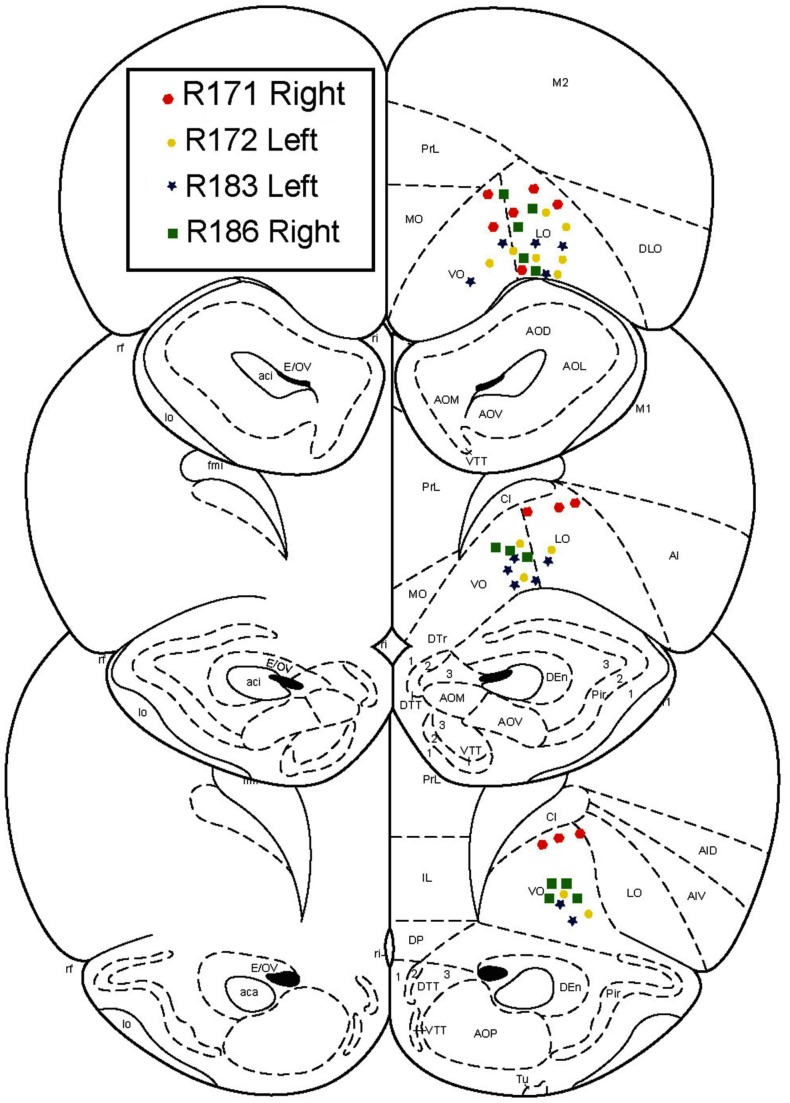
**Histology**. All recordings were confirmed to be in orbitofrontal cortex.

After an initial phase of pre-training and after the rats had reached behavioral criterion, rats were chronically implanted with 14 tetrode-hyperdrives (Kopf). Targets were the ventral OFC, AP +3.5 mm, ML +2.5 mm. Implant sides were alternated on each rat, such that two implants were right centered and two were left centered. Surgical procedures were performed as described previously (Johnson and Redish, 2007). All tetrode locations were histologically verified to lie in the ventral OFC (Figure 2).

### Data acquisition

#### Behavior

Rats were tracked by an overhead camera system via Neuralynx (camera 1). A second camera (camera 2) was centered on T4 to increase positional recording accuracy and to serve as a set spatial window for high-cost choice point (T4) passes. Data for the calculation of orient-reorient behavior, defined as VTE, was taken exclusively from the spatial window defined by the second camera at T4. Before surgery, rats were tracked from an LED attached to a in-house-built backpack; after surgery, rats were tracked from LEDs built into the headstage attached to the implanted hyperdrive.

#### Unit recording

Unit and local field potential activity was monitored as the tetrodes were advanced. Once the tetrodes began to approach ∼4.2 mm in depth, tetrodes were advanced no more than 80 μm per day to allow the tissue to stabilize. Once LFP and units were stable, tetrodes were moved to find the largest possible ensemble.

We recorded neural activity on a 64 channel Cheetah recording system (Neuralynx, Bozeman MT). Session data were recorded to disk and units were identified offline using MClust 3.5. Pre-clusters were formed automatically using Klustakwik. During recordings the position of the rat was tracked using colored LEDs on the headstage. The position was time stamped and recorded in Cheetah by the overhead camera and a second camera centered on T4. A total of 712 cells were recorded. Cell yields were distributed across four rats; R171: 173 cells; R172: 252 cells; R183: 137 cells; R186: 150 cells. Because the recordings were conducted over multiple days it is difficult to rule out that some cells may have been recorded multiple times. Because results were consistent across multiple rats, we remain confident that our results are not due to re-sampling. Analyses that are over-conservative for re-sampling also produce similar results.

### Data analysis

#### Behavior

##### Path linearization

In order to compare multiple sessions of differing paths, the 2D tracking data was mapped to the closest point in a 1D path (Schmitzer-Torbert and Redish, 2004; van der Meer and Redish, 2009). Each path had seven landmarks (Start of Maze, T1–T4, both feeders) with a set number of points between landmarks. The data between each landmark was assigned to a fixed number of spatial bins. Because T4 was centered along the top rail on probe sessions, the path length from T4 to the first feeder on either side was equidistant on all probe sessions.

##### Laps

A lap was defined as a complete loop from the start of the maze to the middle of the bottom rail prior to the start of maze zone. Lap times were defined as the time elapsed from when the rat crossed into the navigation sequence, passed through the feeder zones and finally crossed back into the start of the maze zone. Laps that did not include feeder passes, either correct or incorrect, were excluded. In practice this only occurred when the 40-min session ended with the rat between the start of the maze and T4. On correct laps, the rat was rewarded by 2× pellets at each feeder. On error laps no reward was presented.

##### Vicarious trial and error behaviors (VTE, *zIdPhi*)

**Figure 3:**
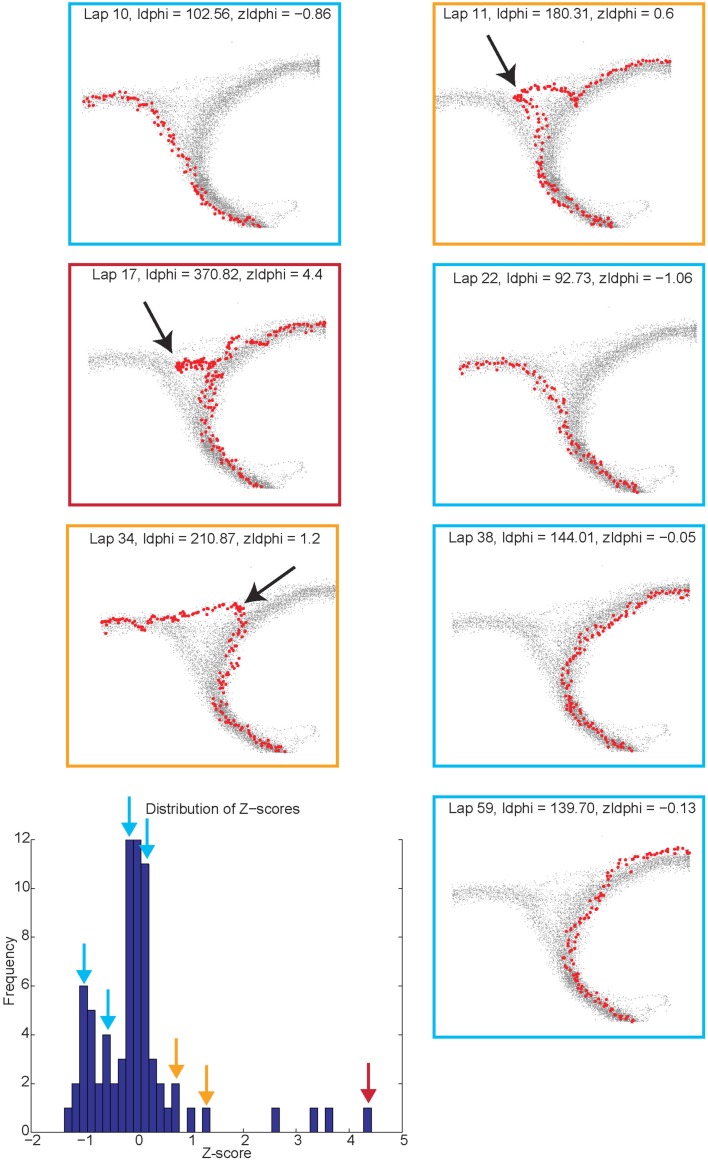
**Identifying vicarious trial and error (VTE) events**. The colored boxes refer to the VTE distribution in the bottom left corner as scored by *zIdphi*. Each pass through camera 2’s field of view is shown in light gray, individual passes (once per lap) through the field of view are highlighted in red. Low *zIdPhi* passes were the most common and demonstrated little behavior indicative of VTE (Blue squares). High *zIdPhi* passes were less common however they demonstrated large, head swings from one direction to the other (yellow and red boxes/arrows on the histogram on bottom left, black arrows on individual behavioral traces from camera 2).

In order to quantify VTE behaviors, we measured the integrated angular velocity (*zIdPhi*) through the choice point pass (Papale et al., 2012). A choice point (T4) pass was defined by entry and exit times through the field of view of camera 2. First, the velocity of the animal was calculated using a modified, discrete-time adaptive window for velocity estimation (Janabi-Sharifi et al., 2000). We used the change in the velocity vectors, *dx* and *dy*, to calculate an angle of motion, and then used the velocity estimation algorithm to calculate the momentary change in angle, *dPhi*. Integrating *dPhi* over the duration of the choice point pass, defined by the box in Figure 1A, resulted in a measure of *IdPhi* which we used to quantify the behavior on a single lap. The *IdPhi* scores were normalized by *z*-scoring across laps for each session for each rat. The *z*-scored measure, *zIdPhi*, was compared across all animals and sessions. This measure proved to be a reliable assessment of the rat’s behavior (Papale et al., 2012; Figure 3). The behavior we observed, previously classified as VTE, was quantitatively defined as *zIdPhi* > 0.5, during which rats reliably demonstrated visible orienting-reorienting behavior (Muenzinger, 1938; Tolman, 1939).

##### Reorientation events

Reorientations were identified at times when the rat performed an abrupt change in direction at T4 (Figure 3, *black arrows*). These events were clearly visible in the tracking data.

#### Single-unit analysis

##### Reward sensitivity

To determine the reward responsivity of a unit, we first calculated a peri-event-time-histogram (PETH) from −1.5 to 3.5 s following feeder triggering using a time step of 100 ms. In order for a neuron to be classified as reward responsive, we compared the number of spikes fired during the 2.5 s following triggering of the feeder (reward delivery, 0–2.5 s) to 500 bootstrapped samples of the same duration aligned to random times throughout the session. If the activity during the reward epoch was significantly different than the bootstrapped samples the cell was classified as *reward responsive*.

#### Decoding

All decoding was performed using a one-step Bayesian decoding method with a time step of 250 ms (Zhang et al., 1998), measuring the probability that the neural ensemble decoded to a given spatial location on the maze. Only cells with >100 spikes and data sets with >14 cells were included in the analyses. We first calculated the linearized tuning curves for each cell during each session. Training sets were extracted from steady state performance. To control for tautology, any test sets used were excluded from the tuning curves in the training sets.

##### Shuffled control data

In order to ensure that the non-local decoding seen in the results does not arise from random firing, we tested our decoding algorithm using tuning curves derived from actual firing patterns and shuffled spike trains. Shuffled spike trains preserves cell identity and the first-order firing statistics of each cell. This allowed us to test whether increased random activity during pauses, at the choice point or at the feeders, could account for the increased decoding to the reward locations.

##### Decoding *p*(Reward)

**Figure 4:**
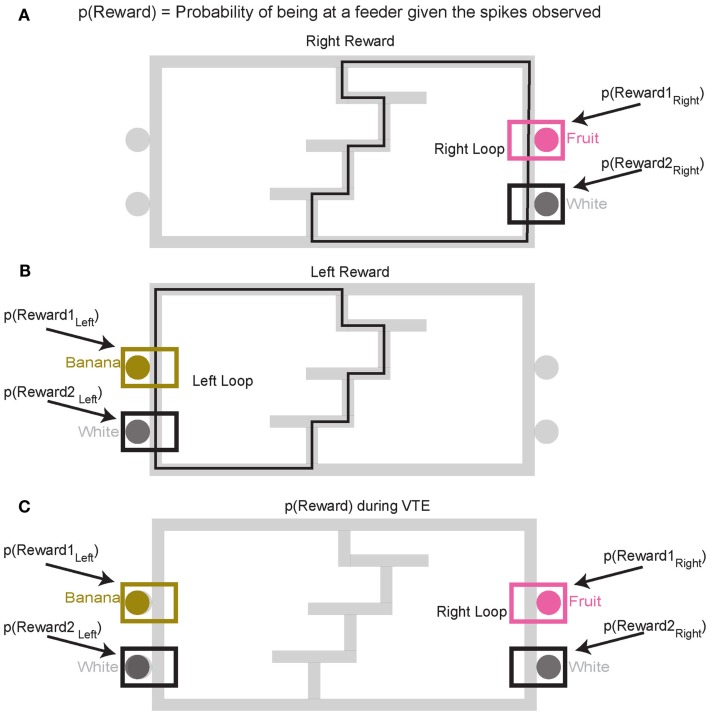
**Decoding locations**. Probability of reward refers to the probability of the rat being at the rewarded site given the spikes that are observed. Because reward sites are fixed in space, a decoding algorithm initially designed to determine predictions in space also reflects the probability of the rat being at a reward site. Since neurons are quantified as being reward responsive based on the presence or absence of reward, the spatial decoding algorithm allows us to the measure the likelihood of receiving reward when the animal is at the reward site, *p*(Reward). It should be noted that we make no claims that OFC represents space. When calculating the probability of reward [*p*(Reward)] for the rightward loop, only the two right feeder locations **(A)** are considered in the calculation of *p*(Reward). Conversely, when considering the leftward loop **(B)**, only left side feeders are considered in the calculation of *p*(Reward). During VTE events, *p*(Reward) is averaged across all four feeder locations **(C)**.

To construct *p*(Reward), each side of the maze was linearized to control for differing lengths in the central portion of the maze (T1–T4) on different days. This produced two separate loops, left and right (Figures 4A,B). Once the maze was linearized, we calculated the spatial tuning curves for all cells on left and right portions of the maze. Because rewards are only delivered at specific locations on the maze, cells which fire primarily in response to reward will drive the spatial location on each loop toward the reward locations. On this task, reward reliably occurs at specific locations on the maze. For example, a cell that fired for banana-flavored reward would fire most on the left loop (Figure 4B). It is important to note that successful decoding to reward locations does not imply that spatial information is encoded in OFC ensembles. Rather we are using a spatial algorithm to provide information regarding the presence or absence of reward. During VTE, decoding was calculated using all cells (see Figure 4C).

##### Decoding at VTE events

Entry and exit times through the T4 choice point were recorded for each pass using the field of view of camera 2. Orient-reorientations were noted. On instances where multiple orient-reorient behaviors were observed, we calculated *p*(Reward) for each event. All cells were used during decoding at VTE events.

##### Counterfactual representations

**Figure 5:**
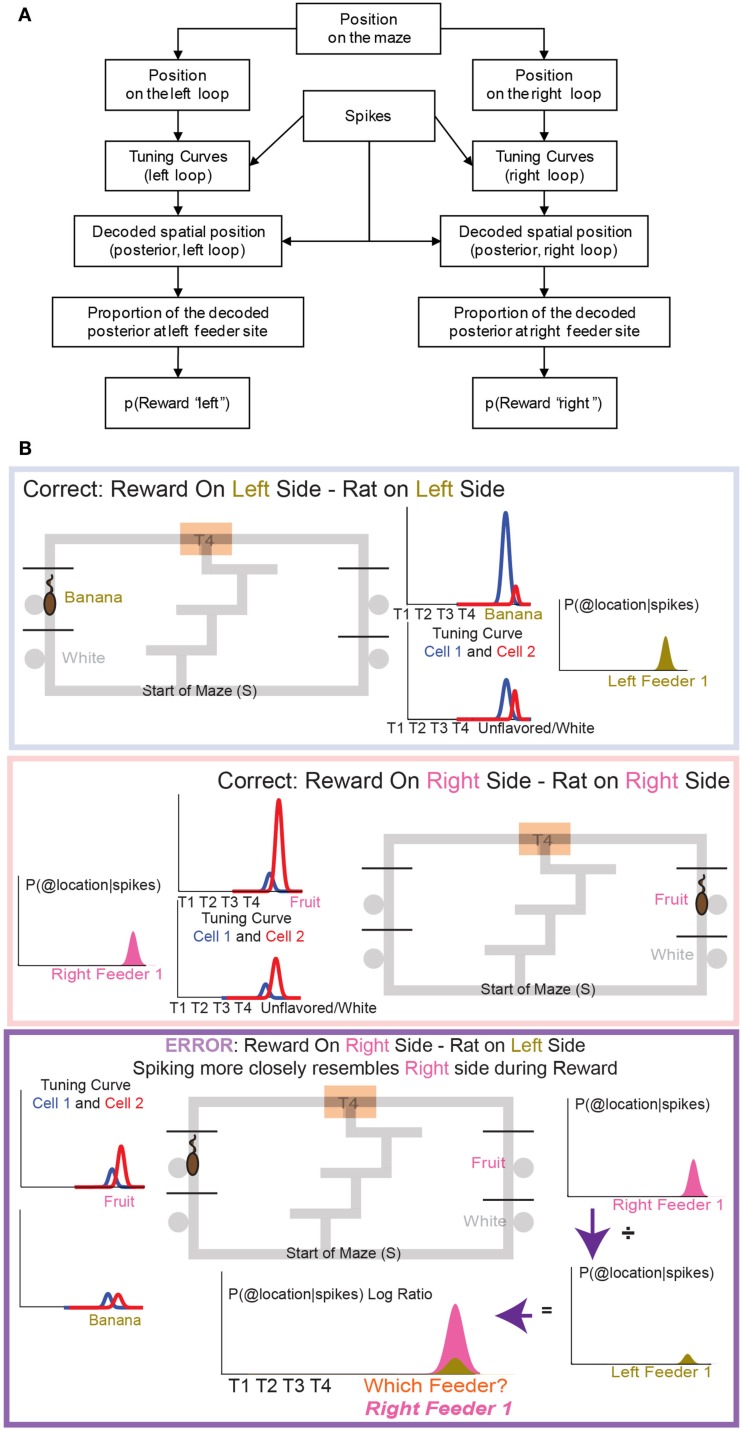
**The decoding process**. **(A)** Any decoding algorithm consists of three parts: (1) a set of tuning curves which defines the expected firing as a function of the variable in question, (2) a set of spikes or firing rates (in order to prevent a tautology, the spikes used in (2) should not be taken from the same set used to generate tuning curves in (1). We accomplish this by a leave-one-out approach in which the tuning curve definition does not include the lap in question), and (3) the posterior probability calculated from (1) and (2). We use two independent decoding processes – one in which the tuning curves are defined from spatial position on the leftward loop only, and the other in which the tuning curves are defined from spatial position on the rightward loop only. Each of these decoding processes provides us with a posterior probability of spatial position around the maze. It is important to note that we do not require that OFC cells be spatial in order to derive spatial decoding posteriors. Because rewards are only delivered at specific locations on our maze, cells which fire primarily in response to reward will drive our spatial decoding to those reward locations (the feeder sites). A cell that fires in response to any reward will drive decoding to all of the reward sites on the maze; a cell that only fires in response to banana-flavored pellets will drive decoding to the first-left-feeder, etc. As shown in Figure 7 we have a diversity of cells which respond to subsets of feeders. We define “reward decoding, *p*(Reward)” as the amount of posterior probability that has been spatially located to those feeder sites. **(B)** Calculating the counterfactual. For two cells with differentiable tuning curves, cell 1 and cell 2, we can use the activity of both cells to determine where on the maze the firing rate best represents the location of the rat. In the top panel, cell 1 prefers banana, and is more active at left feeder 1 when the animal receives banana-flavored pellets. Cell 2 does not respond to banana-flavored pellets. To calculate the decoding we combine information from cell 1 and cell 2 and ask where on the maze does this activity represent? If cell 1 is very active and cell 2 inactive, combing that information increases the probability of decoding to left feeder 1, where the rat has received banana-flavored pellets. This represents an increased probability of decoding to the local reward, *p*(Reward_same_). If cell 2 is now active and cell 1 inactive, we would expect that the probability of decoding to reward would now shift to right feeder 1 (fruit) where the rat just received fruit-flavored pellets (middle panel). Again this would increase the probability of decoding to the local reward site, *p*(Reward_same_). Because both cells differentiate between feeders, we can determine the probability of decoding to a non-local reward as well as a local reward. If the rat is at left feeder and does not receive reward, cell 1 no longer increases its activity. Instead cell 2 increases its activity. We again calculate the probability of decoding. Because cell 1 is inactive, we see very small probability of decoding to the local reward site. However, cell 2 increases its activity. As a result the probability of decoding to the-would-have-been rewarded site, increases (i.e., *right* feeder 1/fruit). To compare both these values, we compare the log ratio of the all the activity and the decoded probability on the local side versus all the activity and the decoded probability on the opposite, non-local side and ask, which side has a higher probability?

Because different sets of OFC cells responded to each of the four reward sites, it was possible to measure *p*(Reward) for a given site. As above, spatial tuning curves for the entire maze were defined for each cell for each loop, right and left, and then separate *p*(Reward) measures were taken from the decoded posteriors at each feeder site. During counterfactual calculations only reward responsive cells were used for decoding *p*(Reward) (see Figure 5).

*p*(Reward_same_) was defined as the *p*(Reward) for the side on which the rat currently was located, while *p*(Reward_opposite_) was defined as the *p*(Reward) for the other side. When constructing *p*(Reward_same_) and *p*(Reward_opposite_) we created two training sets, same and opposite. Same side training sets included correct laps from the same loop that the rat was currently on. Opposite side training sets included correct laps from the opposite loop than the rat was currently on. If, for example, a rat was at right feeder 1 and received reward, *p*(Reward_same_) would be defined from correct rightward tuning curves, while *p*(Reward_opposite_) would be defined from correct leftward tuning curves. On error passes we compared *error*, *non-rewarded* passes to the two test sets, *p*(Reward_same_) and *p*(Reward_opposite_). On correct passes we compared *correct*, *rewarded* passes to the two test sets, *p*(Reward_same_) and *p*(Reward_opposite_).

As above, these training sets were selected from correct laps, either all left correct or all right correct and excluded the lap that contained the feeder pass of interest. *Correct passes* were those at which the rat arrived at the correct feeder and received reward; *error passes* were those at which the rat arrived at the wrong feeder and no reward was present. Decoding was stopped once the rat left the feeder site. Correct laps were matched to error laps by randomly selecting either the correct lap that immediately preceded the error lap or the correct lap that immediately followed it. Correct laps immediately following the start of the session and the switch were excluded from the test sets.

## Results

### Behavior

**Figure 6:**
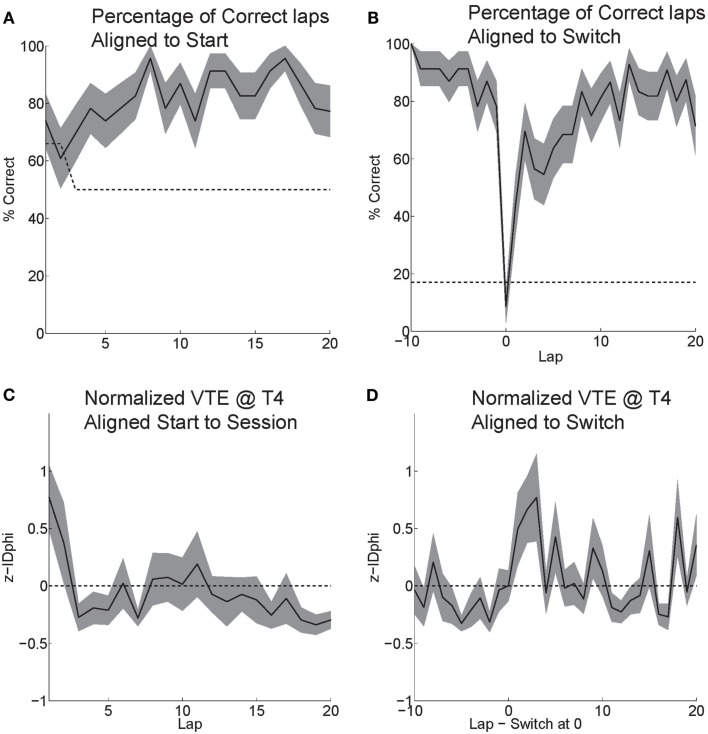
**Behavior on MT-LRA**. **(A)** Rats performed at chance (dashed line) during the first few laps on the task. The rat had a 66% chance to receive food on his first lap because the first lap of an alternation session was always rewarded. As rats discovered the correctly rewarded contingency, their behavior stabilized and the majority of laps were correct (reward received). **(B)** Following the contingency switch, correct accuracy fell below chance (to the expected level the rat would show if it perseverated on the previous first-half-session strategy, dashed line). Rats were not aware of the time of the contingency switch or of the new, correct contingency. **(C)** VTE (*zIdPhi*) from the start of each session by lap. There was a significant effect of early laps on VTE (ANOVA laps 1–5, *F* = 4, *P* < 0.01). **(D)** VTE following the switch was significantly higher than scores prior (Wilcoxon, *P* < 0.01). Comparing the five laps pre switch to the five laps post-switch demonstrated a significant interaction of VTE and lap (ANOVA *F* = 3, *P* < 0.01).

Rats effectively learned the task. During the six probe days with contingency switches, rats started at chance and quickly learned to choose correctly (Figure 6A). Rats maintained a high percentage of correct laps until the change in reward contingency. Following the contingency switch, the percentage of correct laps dropped below chance and gradually returned to a high percentage of correct laps (Figure 6B).

Initially, as the rats learned the task, VTE was high while the rats determined the correct contingency. As rats learned the task and the percentage of correct laps increased, the amount of VTE (*zIdPhi*) demonstrated at the choice point decreased (Figure 6C). When rats encountered the change in contingency, VTE increased drastically and then decreased back to levels seen during stable, correct performance (Figure 6D).

### Reward responsivity at feeders

**Figure 7:**
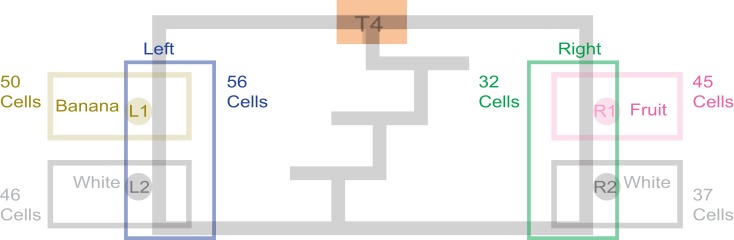
**Number of cells that responded to each different reward type/location**. Many cells responded preferentially for a given reward type. Others responded for a given rewarded side, while some responded for certain combinations of reward sites.

**Figure 8:**
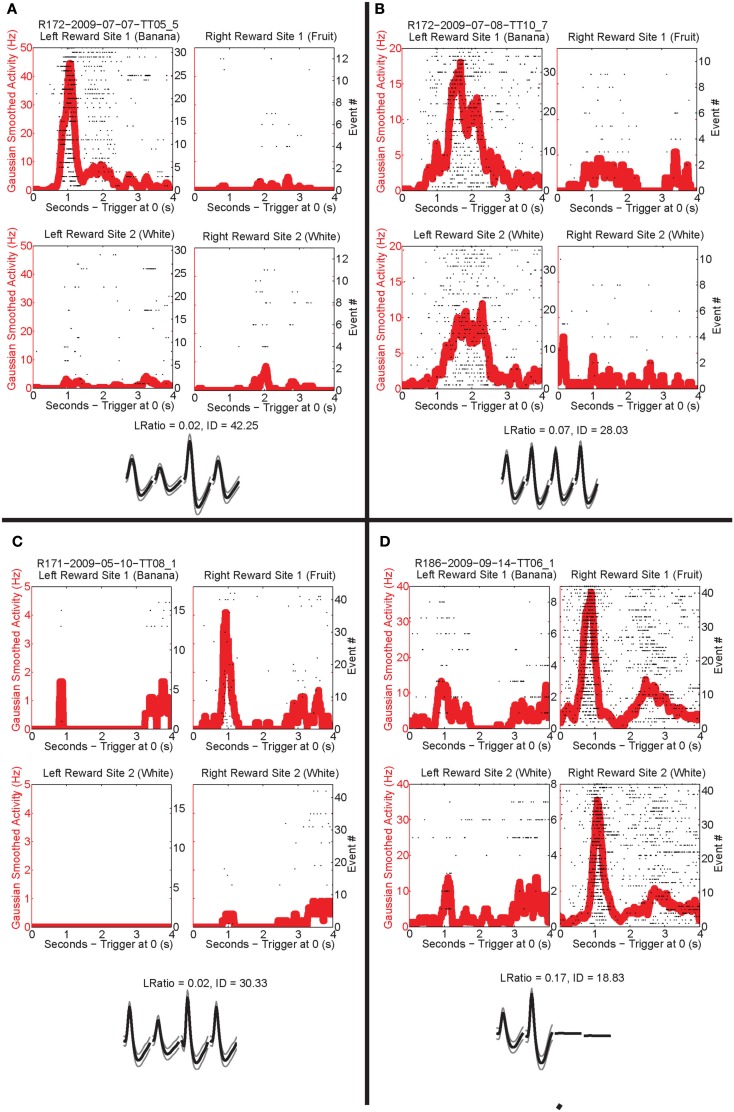
**Individual neurons, different rewards**. Individual neurons prefer left rewards. **(A)** This cell, R172-2009-07-07-TT05_5, preferred Left feeder 1 reward (banana). Very little activity was present at all other reward sites. Panels show rasters at each of the four feeder reward sites for each rewarded feeder pass (event #) with the population density overlaid in the red trace. As indicated by the rasters, this neuron responded significantly more to left feeder 1 (banana). The waveform shows the average waveform of the example neuron, because we are recording from tetrodes, waveforms have four components. The L Ratio and isolation distance indicate that this neuron was well isolated from the other spikes in the session. **(B)** The bottom panels show a different neuron, R172-2009-07-08-TT10_7, which responded to both left feeder rewards (banana left feeder 1 and white, left feeder 2) much more strongly than the right feeder rewards. **(C)** A cell, R171-2009-05-10-TT08_1, that prefers Right feeder 1 reward. **(D)** R186-2009-09-14-TT06_1, which responded more to the right feeder rewards (fruit right feeder 1 and white, right feeder 2).

Past neural recordings in OFC have demonstrated robust reward responses with a variety of specific responses (Schoenbaum and Eichenbaum, 1995a,b; Tremblay and Schultz, 1999; van Duuren et al., 2007, 2009). Some cells responded to different rewards (Figure 7; Table 1). Many cells demonstrated preferential activity for a reward site, some responded more for banana-flavored pellets at Left feeder 1 (Figure 8A) or for fruit-flavored pellets at Right feeder 1 (Figure 8B). Of the 712 cells, 506 (71%) were classified as reward responsive. Cellular reward response dynamics we observed are consistent with prior recordings in this region (Schoenbaum and Eichenbaum, 1995a; Gallagher et al., 1999; van Duuren et al., 2007).

**Table 1:** **Illustrates the different combinations that cells responded for**.

	L1 reward	L2 reward	R1 reward	R2 reward	Left side reward
L1 reward	50				
L2 reward	56	46			
R1 reward	10	10	45		
R2 reward	13	9	32	37	
Left side reward			34	15	
Right side reward	23	19			108

*Each row and column represents a different feeder location. At each intersection point, numbers indicate how many cells responded for the different locations. If the row and column match, then the number reflects the number of cells for one feeder location (L1Reward × L1Reward, 50 cells). If the column and row intersect and are different, then the number reflects the number of cells that significantly responded at both those two sites (R1Reward × L1Reward, 10 cells). Certain cells were active for combinations: 10 cells responded to both L1 Reward and R1 Reward, while 9 cells responded to both R2 Reward and L2 Reward. One hundred eight cells responded at all reward locations*.

Other data have suggested the OFC encodes value during decision processes (Padoa-Schioppa and Assad, 2006, 2008). Given that an individual rat would be expected to have a preference for one food over the other, if the OFC cells were encoding value, we would expect all of that animal’s cells to prefer one food over the other. As shown in Table 2, cellular firing preferences within animal were equally divided between sides. This suggests that the reward responses included sensory information. This interpretation is consistent with recent evidence that OFC represents the sensory aspects of rewards in a current state rather than value and is necessary during model-based decision making (Roesch et al., 2007; McDannald et al., 2011, 2012; Takahashi et al., 2011).

**Table 2:** **Identifies the number of cells, within rat, that responded explicitly to either Left feeder 1 reward (banana) or Right feeder 1 reward (fruit)**.

Rat	Feeder L1	Feeder R1
R171	9	8
R172	14	19
R183	6	6
R186	21	12

### Reward representations during VTE

Previous evidence has suggested that evaluative decision making occurs during orienting/reorienting behaviors, quantified as VTE (Johnson and Redish, 2007; van der Meer and Redish, 2009; Krajbich et al., 2010). If evaluative processes necessary for deliberation are occurring during VTE, and if reward expectations are being modified or generated during this deliberative process, we should expect to see these expectations reflected in OFC activity.

**Figure 9:**
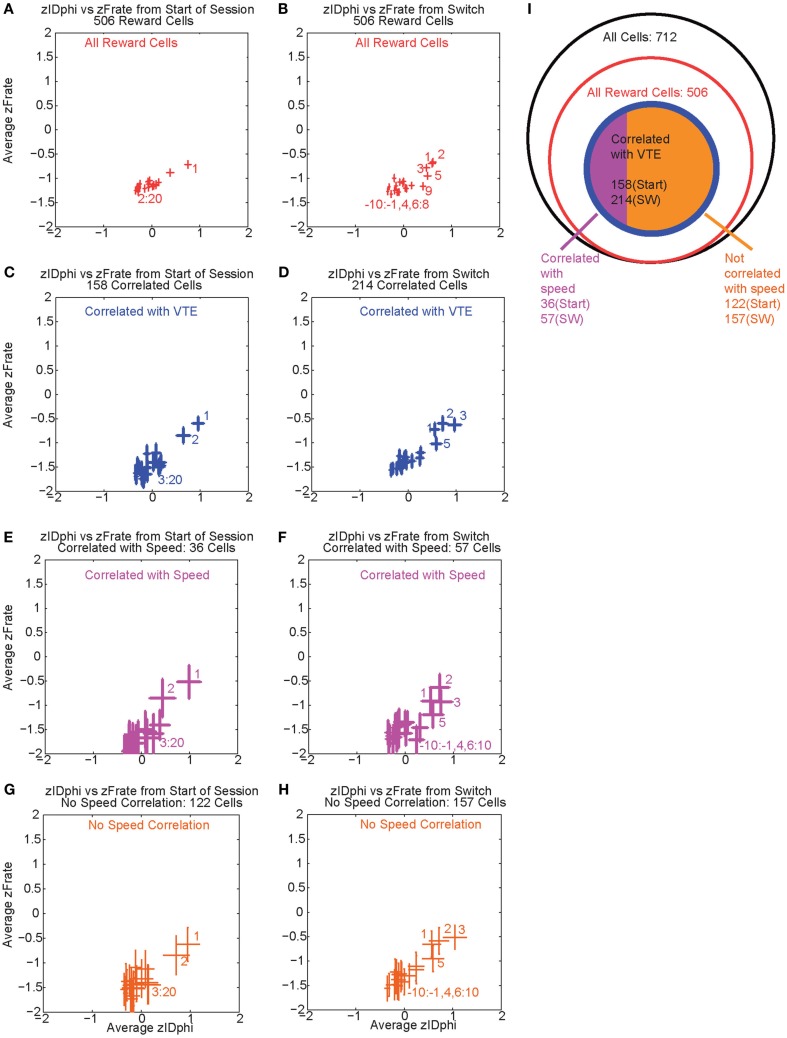
**Firing rate and *zIdphi* relationships**. For each reward responsive cell, the average firing rate through a pass across T4 was calculated. A total of 506 cells responded significantly to reward. We then *z*-scored that firing rate distribution for each cell, producing a *zFRate* measure for each cell for each pass. Each panel shows the average *zFRate* across cells for each lap as a function of *zIdPhi* for that lap. Left panels used lap numbers aligned to the start of the session. Right panels used lap numbers aligned to the switch in reward contingency. For many of these cells, so much activity was present at the reward site relative to the rest of the maze, that the zFiring rate was negative at all locations, even at the choice point, even when the cell fired extra spikes at the choice point and not elsewhere. The shift in *z*-scores during VTE identified that reward responsive cells increased their firing rate, but not to the same level as seen at the reward locations. The top panels **(A,B)** shows the average *zFRate* versus *zIdPhi* for all reward responsive cells (506 cells). The next row of panels **(C,D)** shows the average *zFRate* versus *zIdPhi* for all cells that had a significant correlation between individual firing rate and *zIdPhi* (158 cells aligned to start of the session, 214 cells aligned to contingency switch). Of the reward responsive cells correlated with *zIdPhi*, some cells were also correlated with the speed of the animal during the choice point pass. The third row of panels **(E,F)** shows the average *zFRate* versus *zIdPhi* for all cells that were also correlated with speed (36/158 cells aligned to start of the session, 57/214 cells aligned to contingency switch). The bottom row of panels **(G,H)** shows the average *zFRate* versus *zIdPhi* for those cells not correlated with speed (122/158 cells aligned to start of session, 157/214 cells aligned to contingency switch). The diagram on the right **(I)** depicts the total number of cells (black), the number of reward responsive cells (red), the number of reward responsive cells correlated with *zIdPhi* (blue), and finally the number of reward cells correlated with *zIdPhi* and speed in purple and the number of reward cells correlated with *zIdPhi* but *not* speed in orange.

Over the first 20 laps, 158 of 506 reward responsive cells demonstrated a significant individual firing rate correlation with VTE (Figures 9A,C). Of those 158 cells, 36 were also correlated with speed (Figures 9E,G). Even excluding the cells correlated with speed, many cells continued to show a relationship between *zIdPhi* (VTE) and reward firing while the rat was at T4, indicating that speed could not explain the excess neural activity in reward cells during VTE.

As previously stated, VTE reappeared after the contingency switch. To test if VTE and firing rate were still correlated after the switch, we again calculated the individual regressions for the *z*-scored firing rate of each reward responsive cell against *zIdPhi* by lap. Again, there was a strong relationship between VTE and firing rate, driven in large part by the first few laps post-switch, when VTE was high (Figure 9B). Following the switch in reward contingency, 214 reward responsive cells displayed a significant increase in normalized firing rate with VTE. Of those 214 cells, only 57 were correlated with speed (Figures 9D,F,H). These correlations imply that OFC reward responsive cells increased their firing rates during VTE. This effect could not be explained as simple correlations with speed. (See Figure 9I for a summary of the cells with firing rates correlated with VTE).

**Figure 10:**
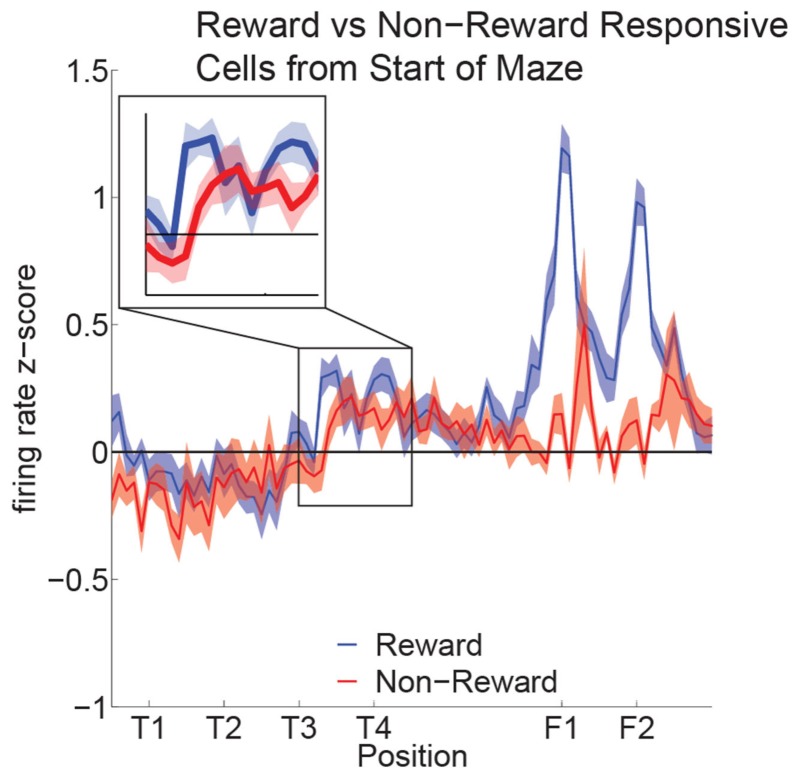
**Increased activity of reward cells at the choice point**. Reward responsive cells showed significantly higher firing rates than non-reward responsive cells at T4 as well as at F1,F2 (ANOVA *F* = 5.3, *P* = 0.03; ANOVA *F* = 23, *P* < 0.01). We compared the normalized activity of reward responsive cells at salient maze locations by linearizing the tracking data from each session and assigning a set number of points between landmarks (Schmitzer-Torbert and Redish, 2004; van der Meer and Redish, 2009). A two-way ANOVA with lap (laps 1–10) and location on the maze (T1 to halfway to F1) as factors demonstrated a significant variation in firing rate across the navigation sequence (*F* = 5.6, *P* < 0.01). A Tukey *post hoc* comparison indicated that the activity at T4 was higher compared to other portions of the navigation sequence (T4: Mean = 0.44, Std = 0.07; Avg T1–T3: Mean = −0.08, Std = 0.09).

Reward cell activity at T4 was significantly different from non-reward cells (Figure 10). Previous research has indicated that ventral striatal reward cells show a similar phenomenon (van der Meer and Redish, 2009). van der Meer and Redish (2009) found that this increased activity translated into an increased decoding to reward locations under a Bayesian decoding analysis. In order to determine whether the increased firing in OFC in Figure 10 entailed a representation of the reward, we determined the extent to which OFC ensembles decoded to reward locations during VTE.

**Figure 11:**
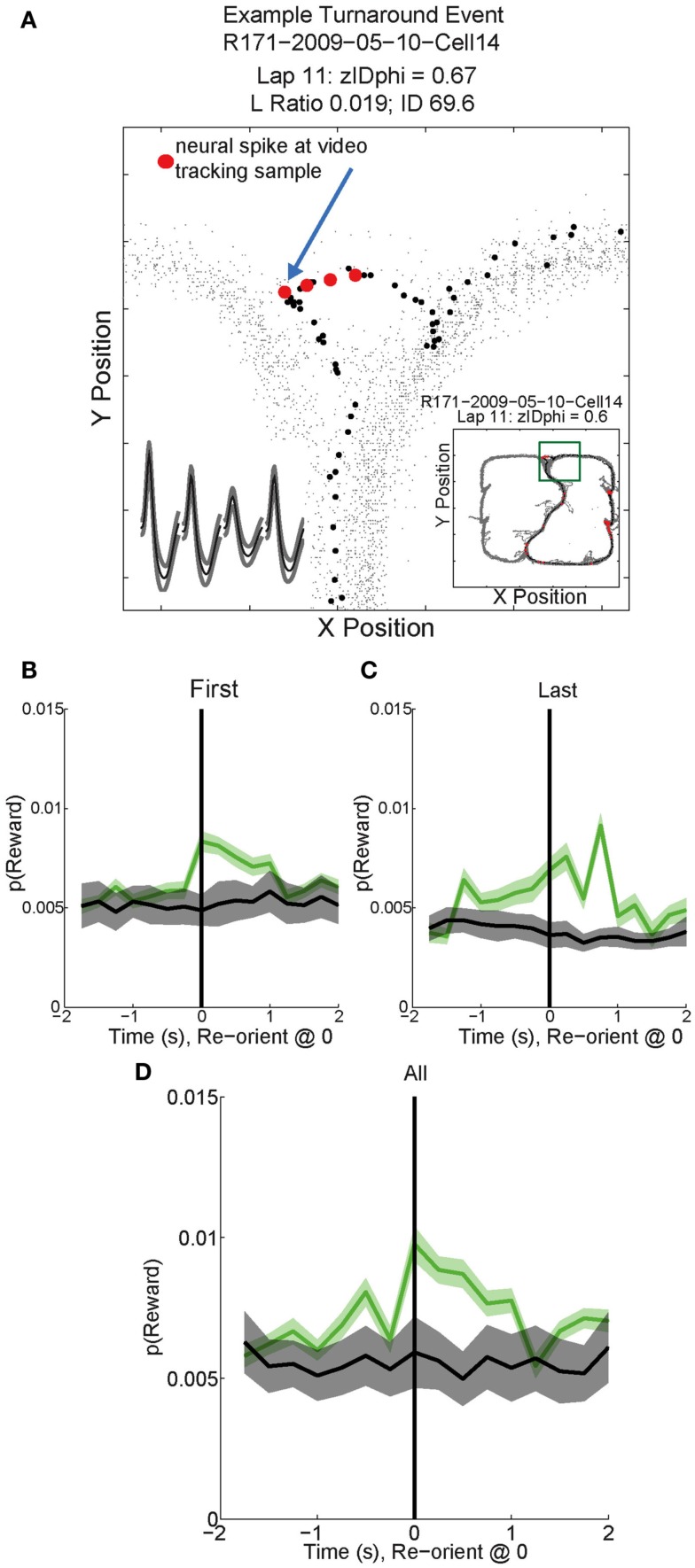
**Example Activity and decoded *p*(Reward) during VTE events**. **(A)** This particular reward responsive cell spiked multiple times during VTE. The inset shows the average waveforms of this cell for each channel of the tetrode. The behavior demonstrates the typical “head sweep” seen during a VTE event (see Figure 3). The red dots indicate neural spikes which are overlaid on top of the behavioral tracking data. The current lap is denoted by black dots. Reorientation is identified by the peak deviation in the pass, indicated by the blue arrow. On many sessions, multiple reorientation events occurred at the choice point. For this figure, *p*(Reward) was calculated using decoding based on all cells. *p*(Reward) was defined as the average posterior probability at each of the reward locations on the maze. Both left feeder and right feeder locations were included as part of the average to calculate *p*(Reward). Average *p*(Reward) peaked immediately after the turnaround (green) on the **(B)** first, **(C)** last, and **(D)** all reorientations at the choice point. We performed a control by shuffling [gray trace in **(B–D)**; *n*Boot = 500] the interspike intervals for each cell and re-calculating the decoding using the unshuffled tuning curves. This control determined that random firing would not reliably decode to reward locations.

As can be seen in Figures 11B–D, *p*(Reward) increased following reorientation events, then decreased as the rat left the choice point and progressed toward the feeders. Individual reward responsive cells showed increased activity during VTE events, as illustrated by the sample cell in Figure 11A. Interestingly, *p*(Reward) decoding to a specific reward site was not seen. Instead reward representations remained general. The non-specific increase in *p*(Reward) seen following reorientation suggests that once the rat has made a decision, his reward expectation reflects the potential for reward rather than an explicit reward such as banana or fruit.

An alternative explanation for increased representation of reward during VTE behaviors could be activity related to the previous lap (which was often an error). Previous reports have indicated that signals in OFC relate to the reward on the previous trial (Sul et al., 2010). On our task VTE does increase during similar laps that errors do (early laps and again after the switch; Blumenthal et al., 2011), however there was no direct relationship between VTE and error trials (comparing VTE after errors to VTE after matched/following correct laps, Wilcoxon, *P* = 0.1329).

### Representations of disappointment and counterfactuals in OFC

**Figure 12:**
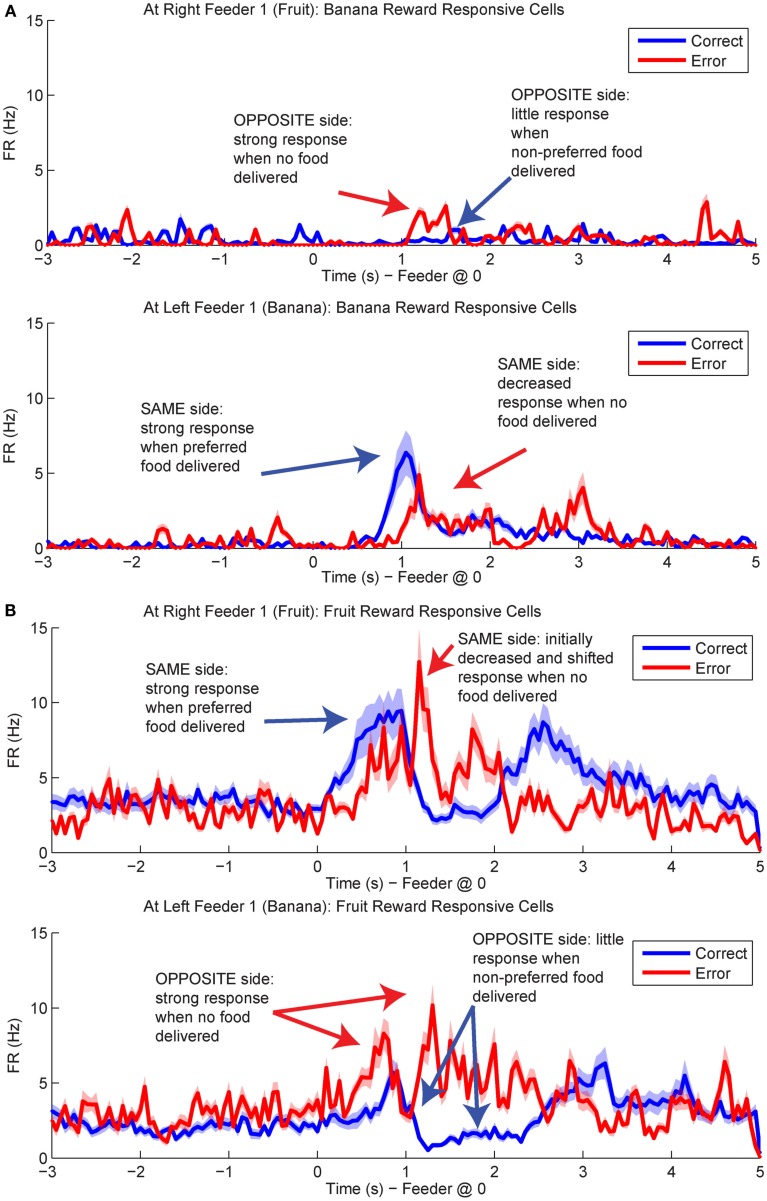
**Population responses during errors**. The average spike population density was calculated for all cells that preferred Left Feeder 1 (Banana) or Right Feeder 1 (Fruit) rewards. **(A)** Left-reward/banana preferring cells. The blue line in the lower plot (correct at left feeder) is larger than the blue line in the upper plot (correct at right feeder). However, note that the activity of left-preferring cells is larger on error laps at the right feeder than on correct laps at the right feeder. **(B)** Right-reward/fruit preferring cells. Cells classified as right-reward preferring demonstrated increased firing at the right-reward sites. The blue line in the upper plot (correct at right feeder) is larger than the blue line in the lower plot (correct at left feeder). However, the activity of right-reward preferring cells is larger on error laps at the left feeder than on correct laps at the left feeder (red trace, lower plot).

Population responses of reward cells differentiated between laps in which rats received reward (correct laps) and laps in which they did not (error laps). Interestingly, on error laps population responses were more similar to that usually seen at the opposite feeder. For example, cells that responded to fruit reward receipt (Right Feeder 1), often responded at Left Feeder 1 during errors, when no reward was present (Figure 12). These results led us to investigate how OFC representations changed during the violation of reward expectation. We determined the difference in *p*(Reward) between correct and error laps and compared decoding on the same side loop to the opposite side loop, that is, *p*(Reward_opposite_) was compared to *p*(Reward_same_).

**Figure 13:**
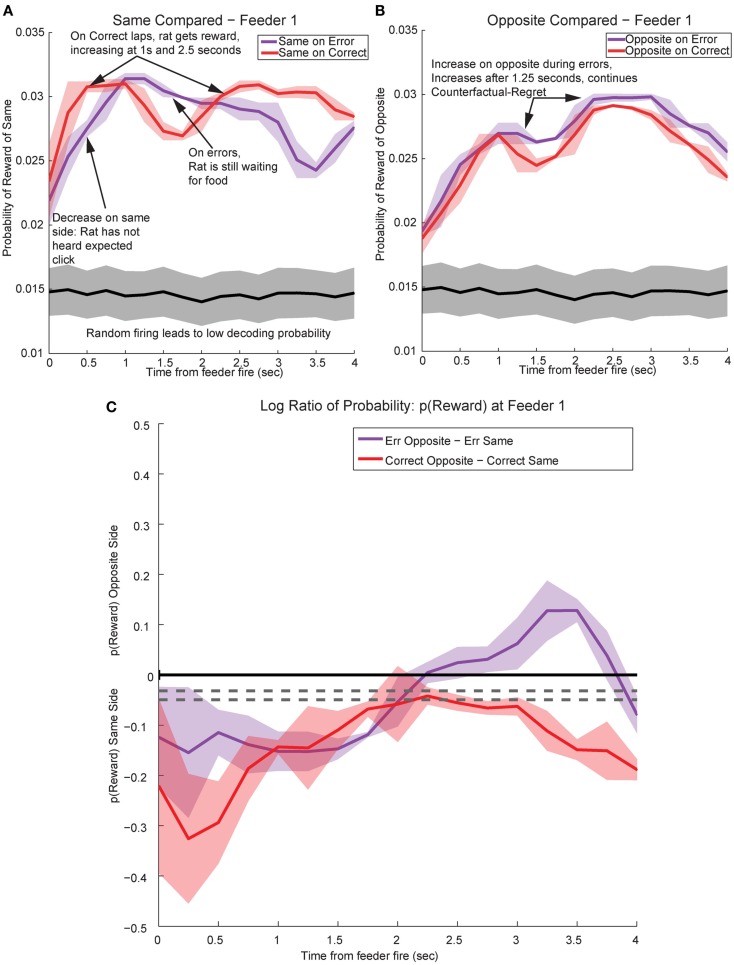
**Decoded *p*(Reward) at Feeder 1 switches sides during errors – disappointment and counterfactuals**. On correct feeder passes, reward followed ∼1.8 s after an audible click (see Figure 1A for zone entry locations). On error passes, no reward is present and the animal does not hear the audible solenoid click. **(A)** In order to determine the presence of disappointment, we examined the difference in *p*(Reward) for the same side loop as a function of whether the choice was correct or an error. As can be seen in the figure, there is a clear shift in the *p*(Reward) on errors (purple trace at ∼1–3 s), when the animal realizes that he has not heard, nor will he hear the solenoid click that he expects and consequently he will not be receiving food. The decrease in local reward representation at 3 s occurs while the rats are pausing at the reward site, several seconds before rats begin to leave the reward site. Additionally, this difference is not a result of random firing in the absence of reward; shuffling the interspike intervals produces a much smaller *p*(Reward) (shown in the gray traces). **(B)** Initially *p*(Reward_opposite_) on error and correct are similar, however, once the rat realizes his error and begins to experience disappointment, neural representations increased to the opposite would-have-been rewarded site. This increase in *p*(Reward) to the opposite would-have-been rewarded side represents the counterfactual signal. **(C)** The log ratio between the local and non-local representations of reward, *p*(Reward_same_):*p*(Reward_opposite_), for correct feeder passes and error passes. Data was smoothed using a 500-ms moving average. Gray lines represent the upper and lower quartiles for shuffled control, based on shuffling interspike intervals and re-calculating the decoding using unshuffled tuning curves. On errors, the log ratio of *p*(Reward) at feeder 1 remained local, [*p*(Reward_same_)* *> *p*(Reward_opposite_)], following arrival at the feeders from 0 to ∼2 s, then switched to a non-local representation [*p*(Reward_same_)* *< *p*(Reward_opposite_)]. In contrast, on correct laps, the log ratio of *p*(Reward) at feeder 1 remained local for the duration of the animal’s pause at the reward site.

By utilizing the two different decoding sets, one for each loop, we were able to compare responses while rats were at the feeders during correct (reward) and error laps (no reward). *p*(Reward_same_) was stronger on correct laps than during errors (Figure 13A). The shift in reward representations on error laps from 1 to 3.5 s seen in *p*(Reward_same_) represented the neural correlate of disappointment; local representation of the reward decreased when the rat finally realized that he was not going to receive reward. This observation of disappointment agrees with the economic definition; disappointment is classified as the realization that available outcome does not match the expected outcome (Bell, 1985; Loomes and Sugden, 1986).

In contrast, *p*(Reward_opposite_) was larger on error laps, when no reward was present, than *p*(Reward_opposite_) on correct laps. This increase indicates that the spiking activity seen during errors better matched the other, would-have-been rewarded side during errors (Figure 13B). The increase in *p*(Reward_opposite_) during errors [∼1.25 s, following the shift in *p*(Reward_same_) during errors] matches the definition of the counterfactual and is consistent with observations of neural representations of counterfactuals in humans; where an alternative, known outcome was better than the received outcome (Bell, 1982; Loomes and Sugden, 1982; Coricelli et al., 2005, 2007).

**Figure 14:**
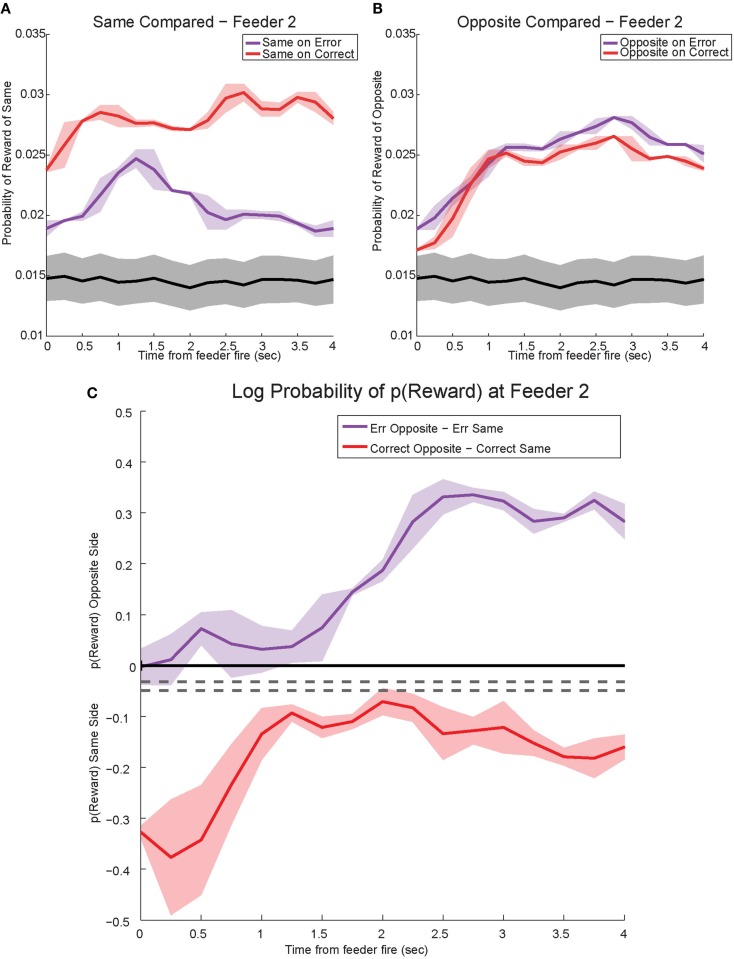
**Decoded *p*(Reward) at Feeder 2**. **(A)** *p*(Reward_same_) on correct was much higher reflecting the reward received. However, during errors *p*(Reward_same_) decreased drastically. **(B)** Initially *p*(Reward_opposite_) on error and correct remained the same. However, once the rat realized he would not be receiving reward, *p*(Reward_opposite_) on errors increased and remained stronger than *p*(Reward_opposite_) on correct. The sustained increase in *p*(Reward_opposite_) during errors, the would-have-been rewarded side, may reflect a planning signal. **(C)** The log ratio between the local and non-local representations of reward, *p*(Reward_same_)*:p*(Reward_opposite_), for correct feeder passes and error passes. Data was smoothed using a 500-ms moving average. Gray lines represent the upper and lower quartiles for shuffled control, based on shuffling interspike intervals and re-calculating the decoding using unshuffled tuning curves. On errors, the log ratio of *p*(Reward) at feeder 2 demonstrated a sustained non-local response, [*p*(Reward_same_)* *< *p*(Reward_opposite_)]. This sustained response to the opposite, non-local side during errors may reflect a planning signal. On correct laps, as seen in Figure 13C, the log ratio of *p*(Reward) at feeder 2 remained local for the duration of the animal’s pause at the reward site.

The shift in *p*(Reward) is best seen by comparing the ratio between *p*(Reward_opposite_) and *p*(Reward_same_), which is best measured as the difference of the logs: log[*p*(Reward_opposite_)] − log[*p*(Reward_same_)]. On correct laps, the difference remains on the same side [i.e., *p*(Reward_same_) > *p*(Reward_opposite_)], which indicates a better representation of the side the animal is on. However, on error laps, the difference is initially located on the same side [i.e., *p*(Reward_same_) > *p*(Reward_opposite_)], but transitions to the opposite side as the rat realizes no food is forthcoming [i.e., *p*(Reward_opposite_) > *p*(Reward_same_)]. Comparing these two changes during *errors*, demonstrates a sustained shift to the would-have-been rewarded side, the counterfactual (Figure 13C). Similar, sustained effects can be seen at the second feeder site (Figure 14).

#### Controls

One potential confound is that in the absence of reward, decoding may become random or drop off. The increased-noise (random firing) hypothesis would predict that decoding would shift away from the representation of the local reward site to become generally uniform across the entire maze. Similarly, the reward prediction error hypothesis would predict that the decoded probability would merely decrease and not increase on the other side. Reward prediction error has been previously seen in OFC (O’Doherty et al., 2003; Sul et al., 2010). Neither of these hypotheses predicts self-consistent representations decoding to the opposite side reward feeder location (Figure 13).

**Figure 15:**
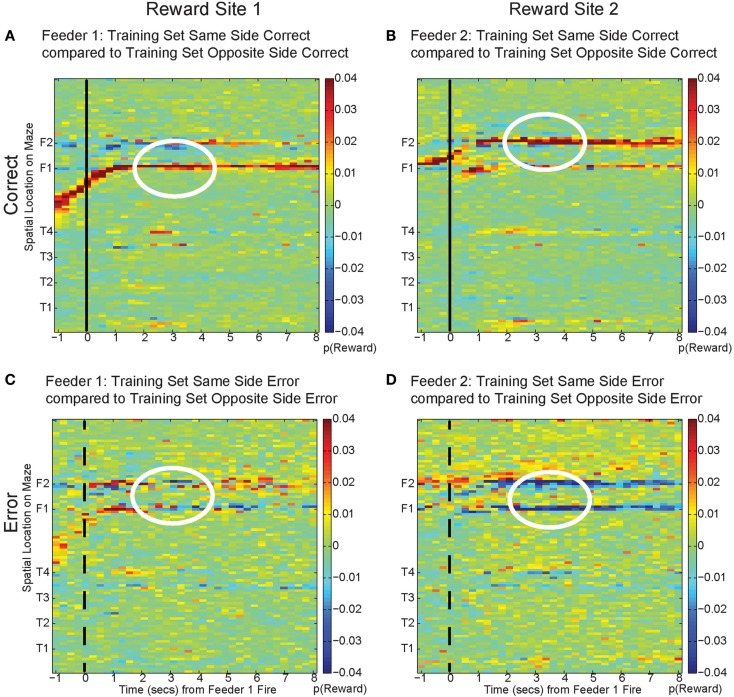
**Decoding across space**. By comparing the log ratio of the decoding generated using all cells and with training sets on same and opposite sides of the rat, we can determine the extent to which neural ensembles reflect the current reward location, the opposite side reward location, or other locations on the maze. Each panel of the figure shows the difference between decoding using tuning curves based on the current side the animal is on and decoding using tuning curves based on the opposite side. As the rat passes the feeder trigger and hears an audible click (solid black line, correct), the rat receives reward and decoding is strongest to the reward site where the rat actually is, as indicated by the strong red color at the correct feeder location [*p*(same) > *p*(opposite), **(A)**: white circle, Feeder 1; **(B)**: white circle, Feeder 2]. This indicates that decoding remains local for the duration of the rats’ stay at the feeder and fails to represent other possible locations on the maze. However, when the rat chooses incorrectly, upon crossing into the zone where he would have heard the reward trigger (dashed black line, incorrect), neural ensembles switch and represent the would-have-been rewarded side, as indicated by the strong blue color at the feeder locations [*p*(same) < *p*(opposite), **(C)**: white circle, Feeder 1 and **(D)**: white circles Feeder 2]. This indicates that neural representations during errors more closely resemble the activity at the would-have-been rewarded feeder. The decoding does not become random; instead *p*(Reward _same_) decreases, while *p*(Reward _opposite_) increases.

To address these potential issues, we compared all decoded locations attained from both correct and error laps. Our decoding algorithm provided posterior probabilities for all possible positions on the linearized maze. By examining the posterior probability at other locations, we can differentiate noise from self-consistent counterfactuals (Figure 15). On correct laps, the differences were significantly positive; the neural activity was representative of the local reward location. This analysis replicated the results seen in Figure 13A, indicating that on rewarded laps, the decoded probability was a better match to the local training sets; *p*(Reward) matched the currently rewarded location of the rat. This analysis also confirmed that on error trials, the decoding better matched the non-local training sets; *p*(Reward) better represented the would-have-been rewarded location. On error laps, the differences were significantly negative. The neural activity was representative of the alternate reward location, not a general change in representation of the entire maze.

The representations did not become random during reward, as would be expected from the increased-noise/random firing hypothesis. Shuffling interspike intervals for the spiking data did not reliably represent reward on the maze (gray traces, Figures 13 and 14), indicating that the increase we see to the would-have-been rewarded side could not be due to an increase in random firing. Nor did *p*(Reward) remain local, as would be expected by a reward prediction error signal. These decoding results indicated that OFC activity was representing the local, rewarded feeder during correct laps and the unchosen (opposite side) feeder during errors.

We can differentiate disappointment from the counterfactual necessary for regret because we are separately measuring the amount of posterior probability assigned to each side independently. When the amount of posterior probability assigned to the same side decreased (i.e., disappointment), it is not necessarily true that the amount of posterior probability assigned to the opposite side would increase. Thus a local, same side decrease represents disappointment, defined economically as the violation of an expectation in the presence of *one* possible outcome (Bell, 1985; Loomes and Sugden, 1986), while an increase in the opposite side representation can be interpreted as a true representation of the alternative reward (i.e., the counterfactual necessary for regret which has been defined as the representation of the alternative outcome when the received outcome does not meet expectations; Bell, 1982; Loomes and Sugden, 1982).

## Discussion

In his poem “Mountain Interval,” the poet Frost (1916) postulated that a traveler faced with a decision pauses to consider possible outcomes, and then compares what is to what could have been. Both of these processes require the representations of information about potential and non-local rewards, a process that relies on OFC (Bechara et al., 1994; Schoenbaum and Eichenbaum, 1995a,b; Tremblay and Schultz, 1999; Camille et al., 2004; Coricelli et al., 2005, 2007; Padoa-Schioppa and Assad, 2006; Platt and Hayden, 2011). In this paper, we report evidence that OFC is involved in both of these processes: (1) During orient-reorient behavior (previously identified as VTE), as rats pause to consider possible outcomes, OFC encodes expectations of reward. (2) During errors, OFC first encodes the disappointment (local) caused by the violated expectation and then transiently encodes the alternative, would-have-been rewarded option (non-local).

### Orientation and reorientation

During early learning, computationally expensive, action-outcome processes attempt to predict reward through a series of what-if scenarios (Daw et al., 2005; Johnson et al., 2007; van der Meer et al., 2012). These scenarios can be evaluated without the direct execution of the action that leads to the outcome. During this vicarious evaluation, hippocampal ensembles represent prospective paths and ventral striatal ensembles indicate the presence of expected reward (Johnson and Redish, 2007; Johnson et al., 2007; van der Meer and Redish, 2009). Here, we report that OFC ensembles reflect the expectation of general reward after reorientation. The general representation of reward during VTE agrees with previous data based on hippocampal representations during VTE. Hippocampal representations of the alternate choices did not reliably represent the direction the animal is facing – an animal could face to the left, but show a sweep of hippocampal representations to the right (Johnson and Redish, 2007). In our OFC data, we did not find reliable reward decoding that differentiates outcomes based on the orientation of the animal.

Other data indicates that OFC representations differentiated uncertainty on a trial by trial basis (Kepecs et al., 2008). It is possible that during VTE, as the rat accesses internal representations of a reward expectation, uncertainty about the upcoming reward modulates the representation of reward and contributes to VTE. Additionally, activity in OFC may reflect some degree of decision confidence as the rat executes the turnaround and proceeds to a possibly rewarded site (Kepecs et al., 2008; Mainen and Kepecs, 2009).

### OFC and ventral striatum

As shown in Figure 11, OFC ensembles decode to represent reward immediately after each reorientation during the VTE process. This suggests that OFC is likely to be involved in expectation following reorientation. In contrast, van der Meer and Redish (2009) found that ventral striatal representations of reward generally *preceded* reorientation. The tasks were identical and these reorientation processes occurred at the same location on similar laps. This suggests a difference between ventral striatal and OFC roles in decision-making processes.

### OFC and reward prediction error

Our data suggest that OFC represents potential reward expectations, and our data are not consistent with OFC representations of reward prediction error. With experience, reward prediction error decreases. However, we did not see evidence for this decrease. Because reward delivery at the two feeder sites on a given return rail were always either both provided (correct lap) or both not provided (error lap), reward prediction error signals would predict no activity at the second feeder site on a given lap. As shown in Figure 8, robust reward-related activity was seen at the second feeder site. In fact, we were able to identify both disappointment signals and counterfactual signals at the second feeder site. While some data suggest the presence of reward prediction error information in OFC (O’Doherty et al., 2003; Sul et al., 2010), other experiments have suggested that OFC more closely tracks outcomes and value rather than prediction error (Daw et al., 2006; Padoa-Schioppa and Assad, 2006; Hare et al., 2008). Recent data suggest that prediction errors in the ventral tegmental area rely on value-state representations from OFC (Takahashi et al., 2011).

### OFC’s contribution to a decision

Previous evidence from Johnson and Redish (2007) has identified that hippocampal representations sweep ahead of the animals location. Additional evidence links normal OFC function to the presence of an intact hippocampus (Ramus et al., 2007). When an animal approaches a decision-point, hippocampal ensembles represent the possible paths. Following the spatial representations, ventral striatal ensembles represent the possible reward that lies at the end of the represented paths. Our current data suggests that OFC represents the expectation of reward following the representation of reward in ventral striatum and the representation of potential paths in hippocampus. This implies that hippocampus likely contributes information regarding the paths to reward concurrently with reward/value information on the upcoming reward from ventral striatum. This information may be combined in OFC to form a state expectation, which includes the relevant sensory aspects of the reward, the reward type and other unique reward properties.

On MT-LRA, because reward sites are at a fixed location, location and reward are confounded. However, if OFC is representing the state of the expected reward, then all salient features of the reward, including the contingency (side) of the reward may be represented. The representation of state characteristics would allow an animal to make decisions using model-based processes (Takahashi et al., 2011; Lucantonio et al., 2012; McDannald et al., 2012; see also Daw et al., 2005; van der Meer et al., 2012). The exact timing of the reward representations in OFC and ventral striatum would be of much interest and could potentially provide useful evidence of a functional dissociation between OFC (state expectation) and ventral striatum (value calculation). Our data indicates that reward expectations peak in OFC after the turn-around point. van der Meer and Redish (2009) found that the reward expectations in ventral striatum peaked before the turn-around point. However, it is important to note that the recordings came from different tasks and slight variations in the task procedures could have affected these timings. Further experiments recording neural ensembles from both locations simultaneously are likely to be fruitful.

### Planning and counterfactuals

There is a close relationship between the ability to plan, which must include a representation of the potential outcome, and counterfactuals, a representation of the alternative that might-have-been. Planning often occurs after violations of expectations and the experience of disappointment or regret. Disappointment arises when situational expectations are violated and these violations are beyond the scope of one’s control (Bell, 1985).

Disappointment (a lack of delivery of expected reward) is inherently aversive (Rescorla and Wagner, 1972), however, disappointment and aversion must be computationally distinct entities because they show different relationships to extinction (Redish et al., 2007) – disappointment has the effect of extinguishing reinforced behaviors while aversion is extinguished by relief. Early economic studies (Bell, 1985; Loomes and Sugden, 1986) defined disappointment explicitly as reward omission. In our task, “disappointment” can be distinguished from the “counterfactual necessary for regret” because disappointment entails the recognition that an expected reward is not going to be delivered, while regret is the recognition that an alternative choice would have produced a better reward (Bell, 1982; Loomes and Sugden, 1982). This requires the evaluation of the current reward, the expectation, and the possible alternatives.

Our data indicate that when the rat discovers his error at the first feeder, OFC representations of reward decrease at the expected reward location, implying disappointment, in conjunction with a distinct, transient increase in the representation of the other, alternative would-have-been rewarded option, the representation of the counterfactual. The strong shift during errors in *p*(Reward_same_) occurred while rats were pausing at the first feeder waiting for reward and then drastically decreased several seconds prior to departure for the second feeder. Therefore we find it unlikely that the decrease in reward representations is related to movement away from the reward site or increased distance from the first reward site. Following the evaluation of the counterfactual, the feedback from the current lap could serve to instruct choice on subsequent laps, which could serve as a planning signal. We find it unlikely that the transient representation at Feeder 1 is a planning signal because the rat still has to go to the second (unrewarded) feeder before proceeding to the next lap. Rats reliably stop and check the second feeder, even when unrewarded. The increased decoding to the would-have-been rewarded side at the second feeder, however, may reflect a planning signal and represent a form of episodic future thinking (Johnson et al., 2007; Peters and Buchel, 2010; van der Meer et al., 2012).

### Violation of expectations

Regret entails a comparison between the expected outcome and a better alternative, which implies a comparison between *multiple* choices (Bell, 1982; Camille et al., 2004). This means that regret requires a representation of a counterfactual, the better alternative. Although the log ratio analysis in Figure 13C cannot differentiate between disappointment and regret, the evidence in Figures 13A,B does differentiate, indicating that the decoding during errors is to the opposite reward location rather than a general diminishment in decoding quality. This implies that the transient representation includes disappointment (local) and the counterfactual (non-local).

The decrease seen in the representations of the local side reflects the absence of reward, a disappointing outcome. This is followed by a transient representation of the known alternatives, an increase in the decoding to the opposite side, and the comparison of what could have been to what was; the counterfactual and the experiencing of regret. Interestingly, the evidence that OFC is required for the generation of a reward prediction error in the ventral tegmental area, may imply that disappointment and regret drive the formation of the reward prediction error elsewhere (Schoenbaum et al., 2011; Takahashi et al., 2011). In human subjects during fictive learning, OFC activity increased when subjects were considering the possible outcomes of their actions in different conditions (Montague and Lohrenz, 2007; Spitzer et al., 2007). Following this hypothesis, reversal learning could be considered to be a form of “learning from one’s regret.” Both humans and animals with OFC lesions are impaired on reversal tasks (Bechara et al., 1994; Damasio, 1994; Dias et al., 1996; Schoenbaum et al., 2002). Additionally, human subjects with OFC lesions do not exhibit the negative emotional arousal associated with the experience of regret (Camille et al., 2004; Coricelli et al., 2005). Regret and disappointment could contribute to the maintenance of reward expectations in OFC for a situation or a given model-based representation (McDannald et al., 2011, 2012; Schoenbaum et al., 2011; Lucantonio et al., 2012).

In humans, value representations of alternative outcomes (counterfactuals) activate OFC (Coricelli et al., 2005, 2007). The timing of these counterfactual representations agrees with experiences of regret and is correlated with fMRI BOLD activation in OFC and anterior hippocampus among other structures (Coricelli et al., 2005, 2007; Platt and Hayden, 2011). Which leads to the question: are rats capable of experiencing regret? Regret requires the comparison between an actual outcome and a counterfactual outcome that would have been the result of an unchosen action (Bell, 1982; Camille et al., 2004; Coricelli et al., 2005, 2007). There may be some evidence of causal reasoning in rats, though this evidence is contentious (Blaisdell et al., 2006). On our task, when a rat chose incorrectly and arrived at non-rewarded feeder sites, neural ensembles in OFC representing reward switched to represent reward on the opposite, rejected side, implying that rats can at least represent the counterfactual necessary for regret.

## Conflict of Interest Statement

The authors declare that the research was conducted in the absence of any commercial or financial relationships that could be construed as a potential conflict of interest.
